# Supplementary material for: Genetic and Pharmacologic Targeting of Eya3 in Macrophages Drives Anti-Tumor Immunity in Triple-Negative Breast Cancer
Source: bioRxiv. 2026 Jul 23:2026.07.22.739910. Preprint. [Version 1] doi: 10.64898/2026.07.22.739910 (PMC13419451; doi:10.64898/2026.07.22.739910)
Supplement: Supplementary file 1 [file NIHPP2026.07.22.739910v1-supplement-1.pdf]

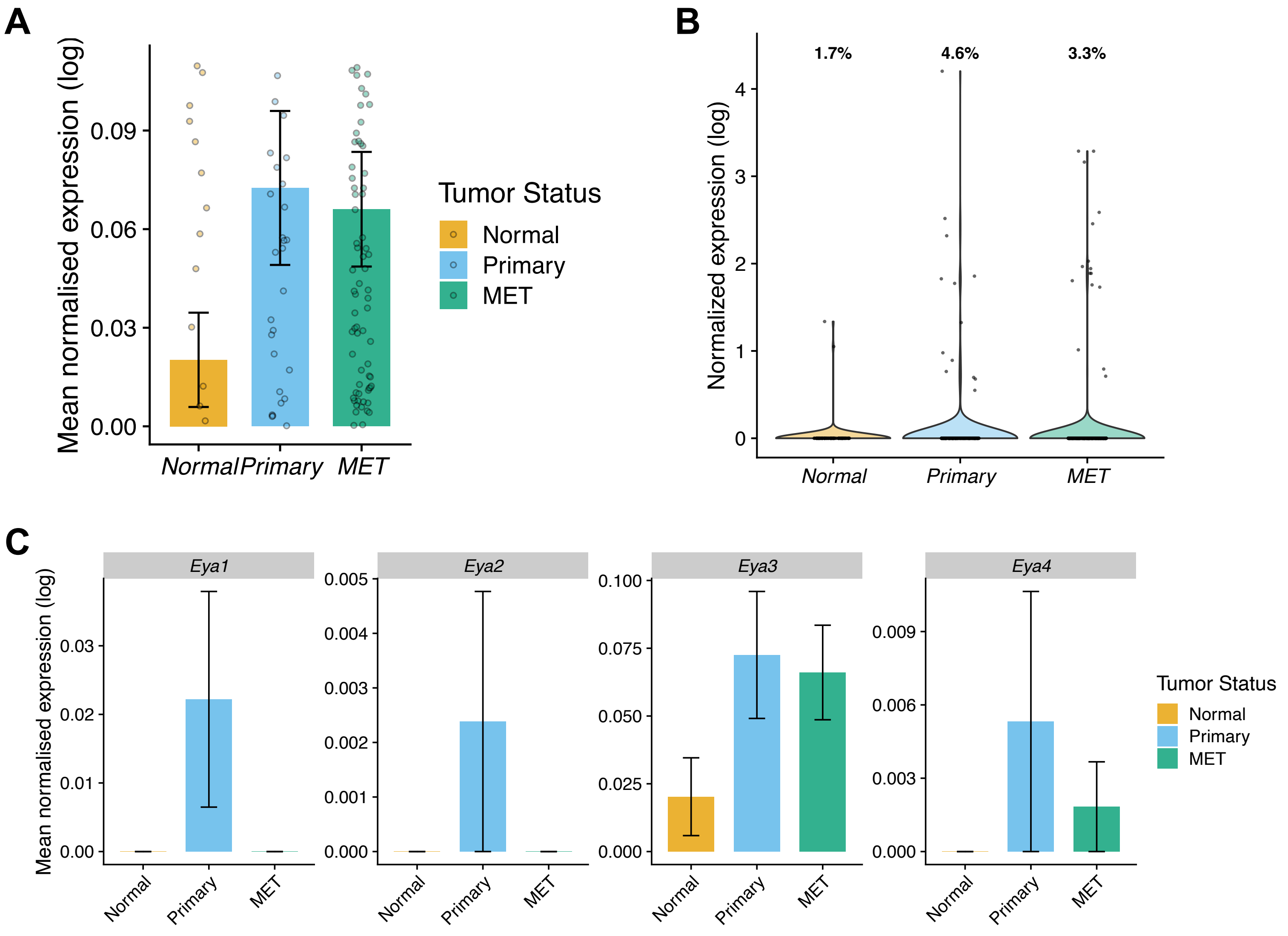

Supplemental Figure 1. ***Additional plots for the quantification of Eya proteins in macrophages in murine normal breast, primary breast tumor, and lung metastasis.*** A) Macrophage Eya3 expression in normal breast, primary tumor, and lung metastasis as dot plot and B) violin plot. C) Expression of all Eya proteins in macrophages from murine normal breast, primary breast tumor, and lung metastasis. All data from GSE231915.

A

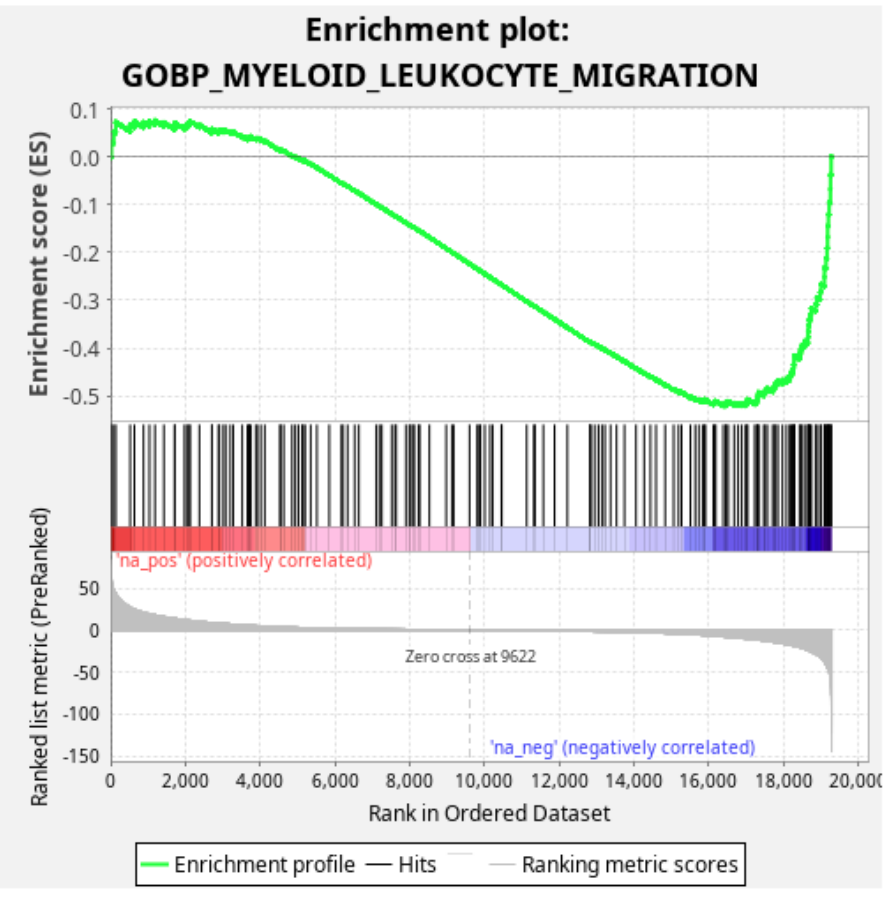

NES = -1.72
FDR q-value = 0.0216

B

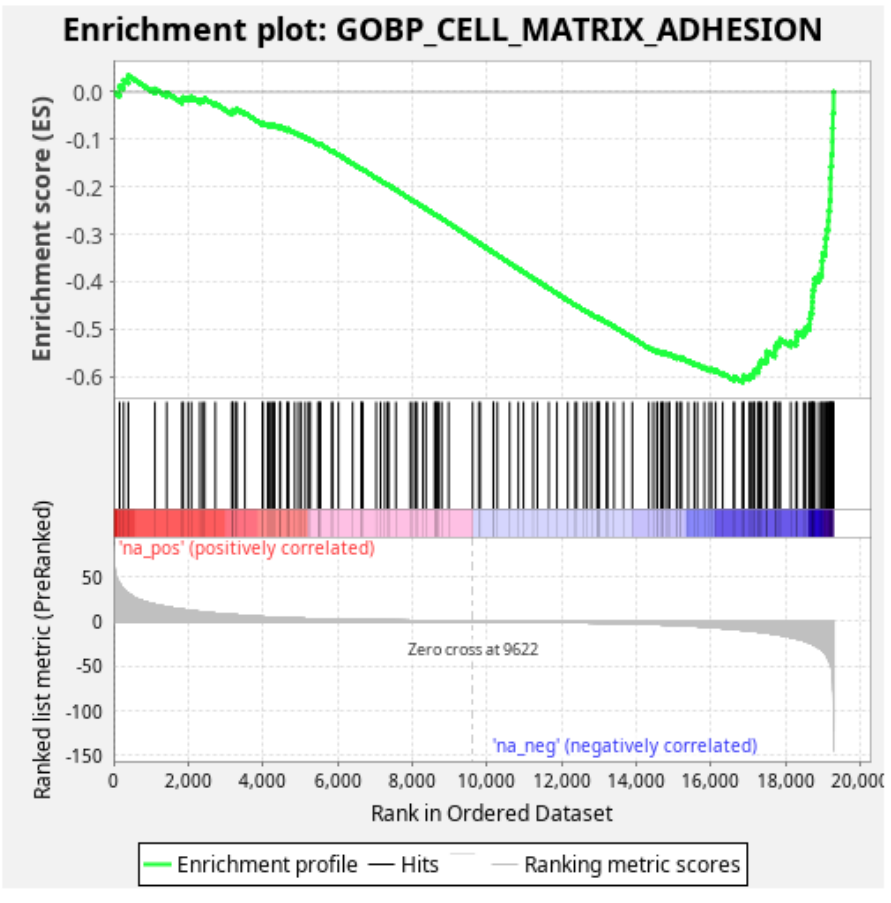

NES = -2.03
FDR q-value = 0.0002

C

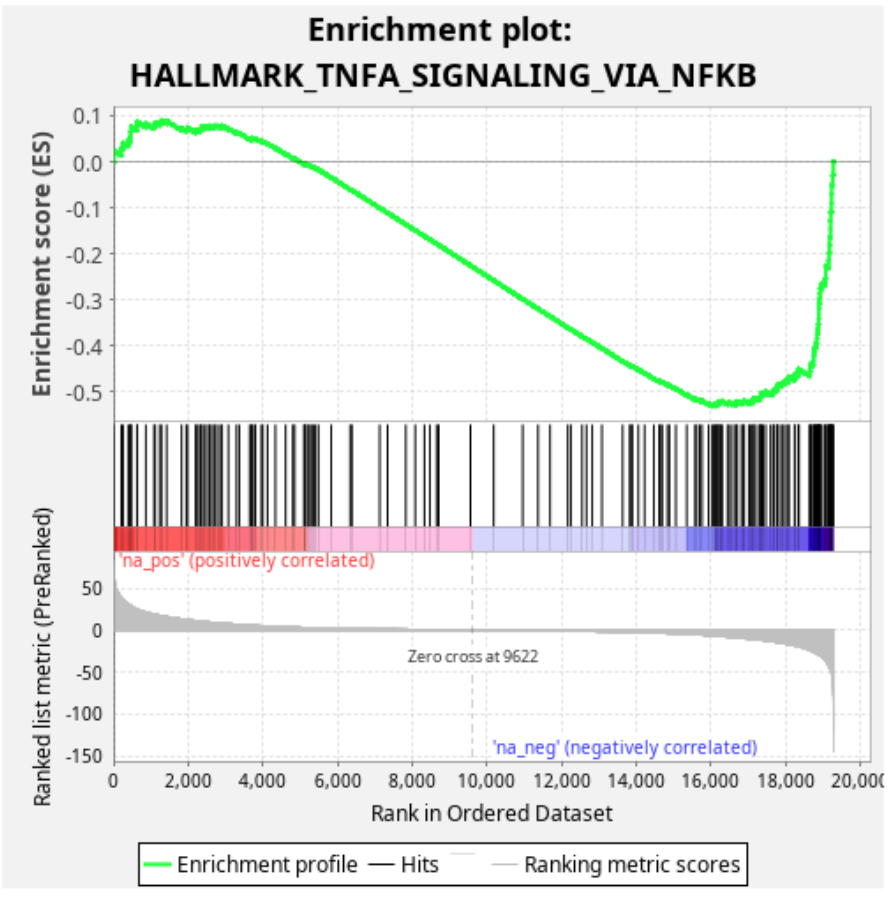

NES = -1.75
FDR q-value = 0.0169

D

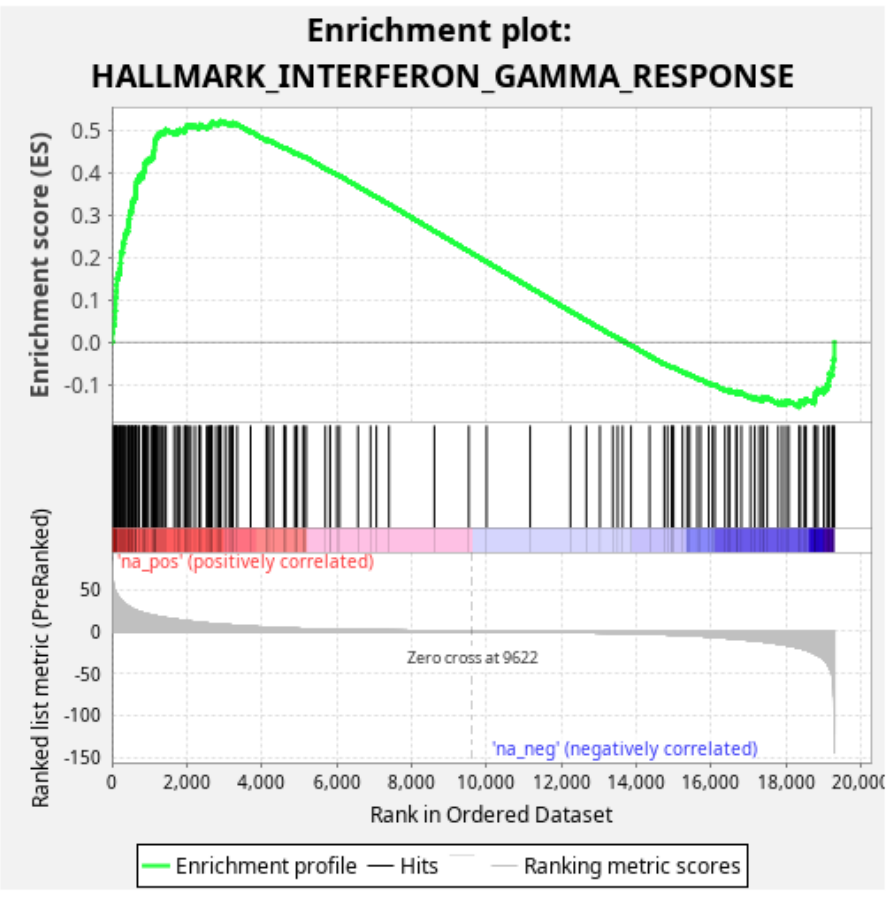

NES = 1.70
FDR q-value = 0.0095

E

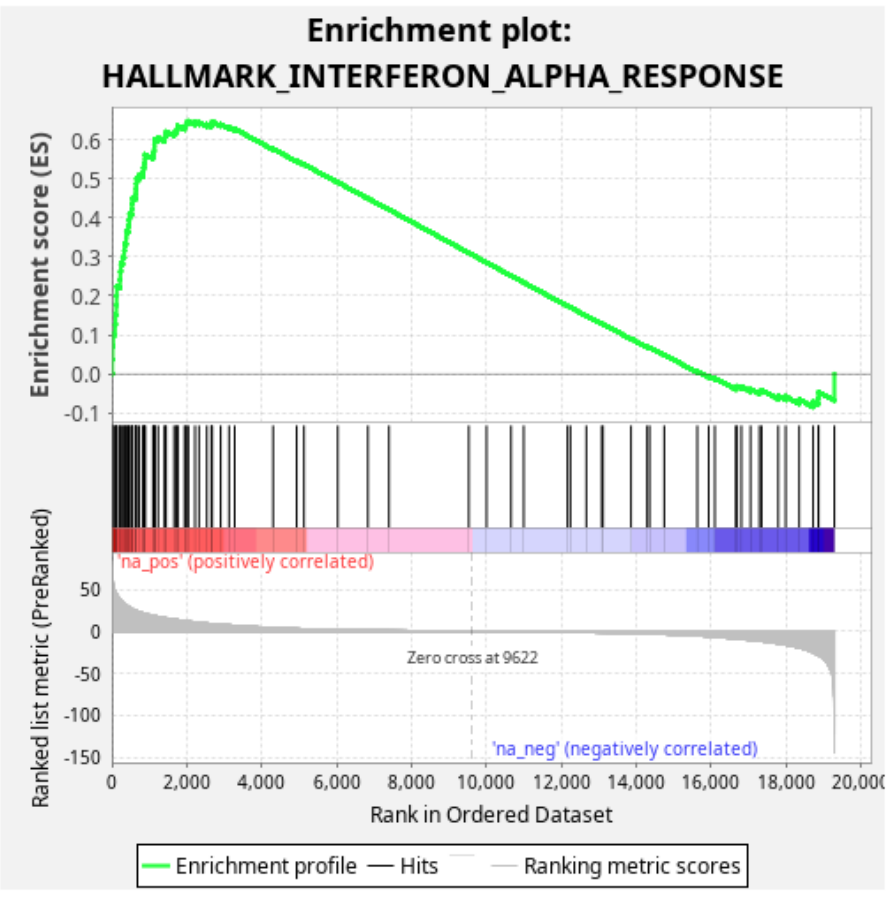

NES = 1.96
FDR q-value = 0.0001

F

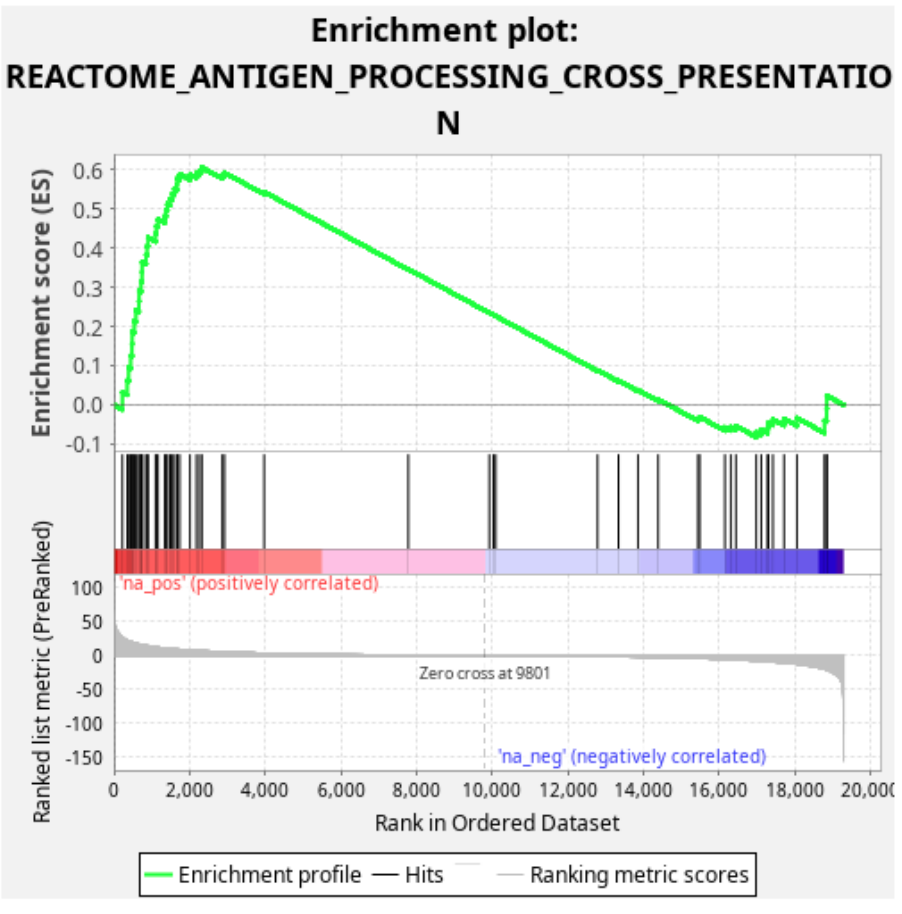

NES = 1.75
FDR q-value = 0.0062

G

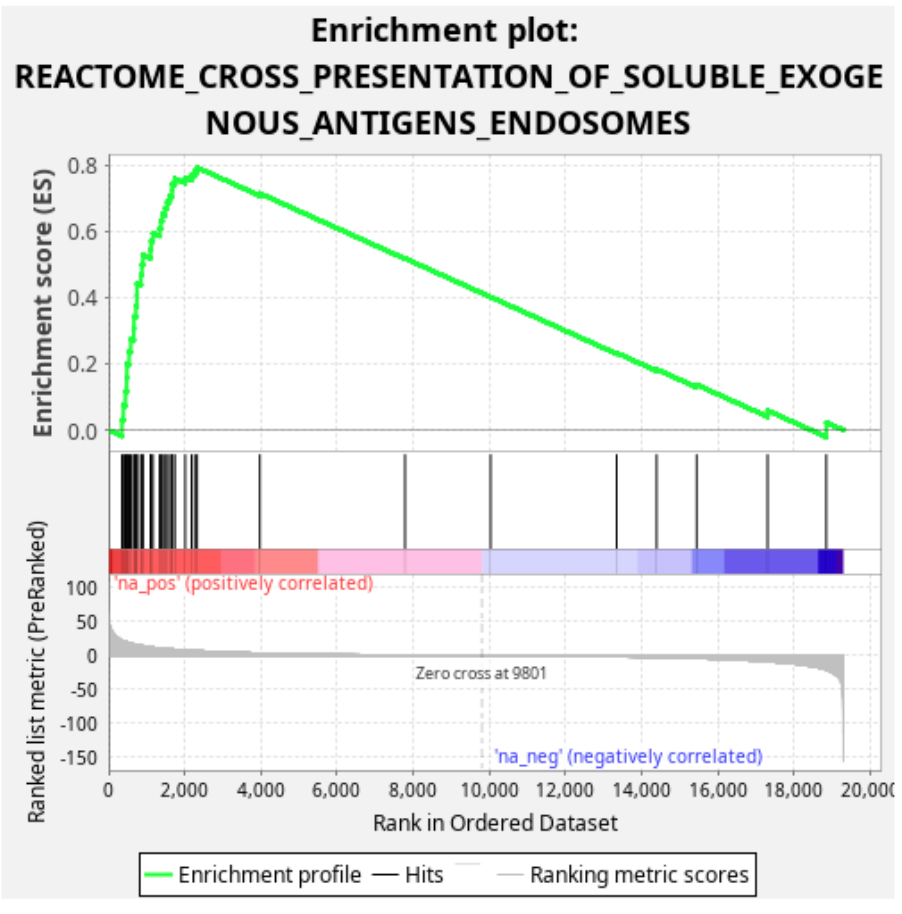

NES = 2.13
FDR q-value = 0.0001

H

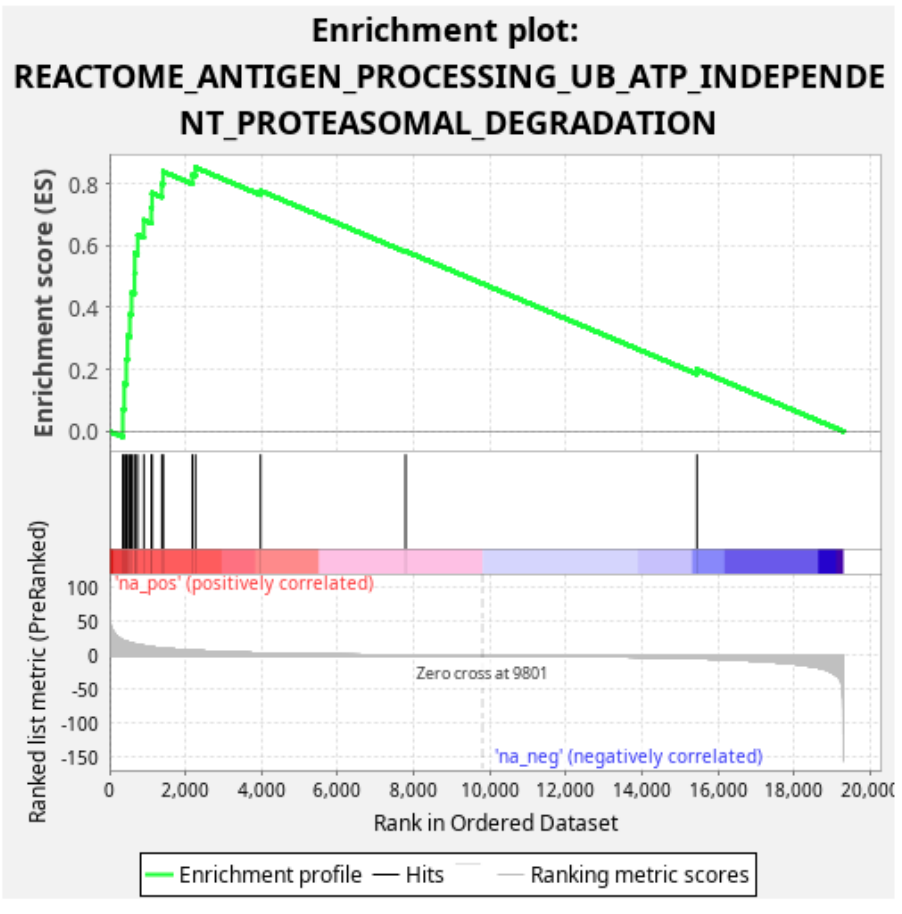

NES = 1.98
FDR q-value = 0.0001

Supplemental Figure 2. ***GSEA plots of pathways relevant to macrophage function from RAW264.7 shSCR and shEYA3 KD bulk RNA-seq.*** GSEA plots comparing RAW264.7 shEYA3 KD2 vs shSCR RNA expression: A) Myeloid Leukocyte Migration B) Cell Matrix Adhesion C) TNFA Signaling via NFKB D) Interferon Gamma Response E) Interferon Alpha Response F) Antigen Processing and Presentation G) Cross Presentation of Soluble Exogenous Antigens Endosomes H) Antigen Processing UB ATP Independent Proteasomal Degradation

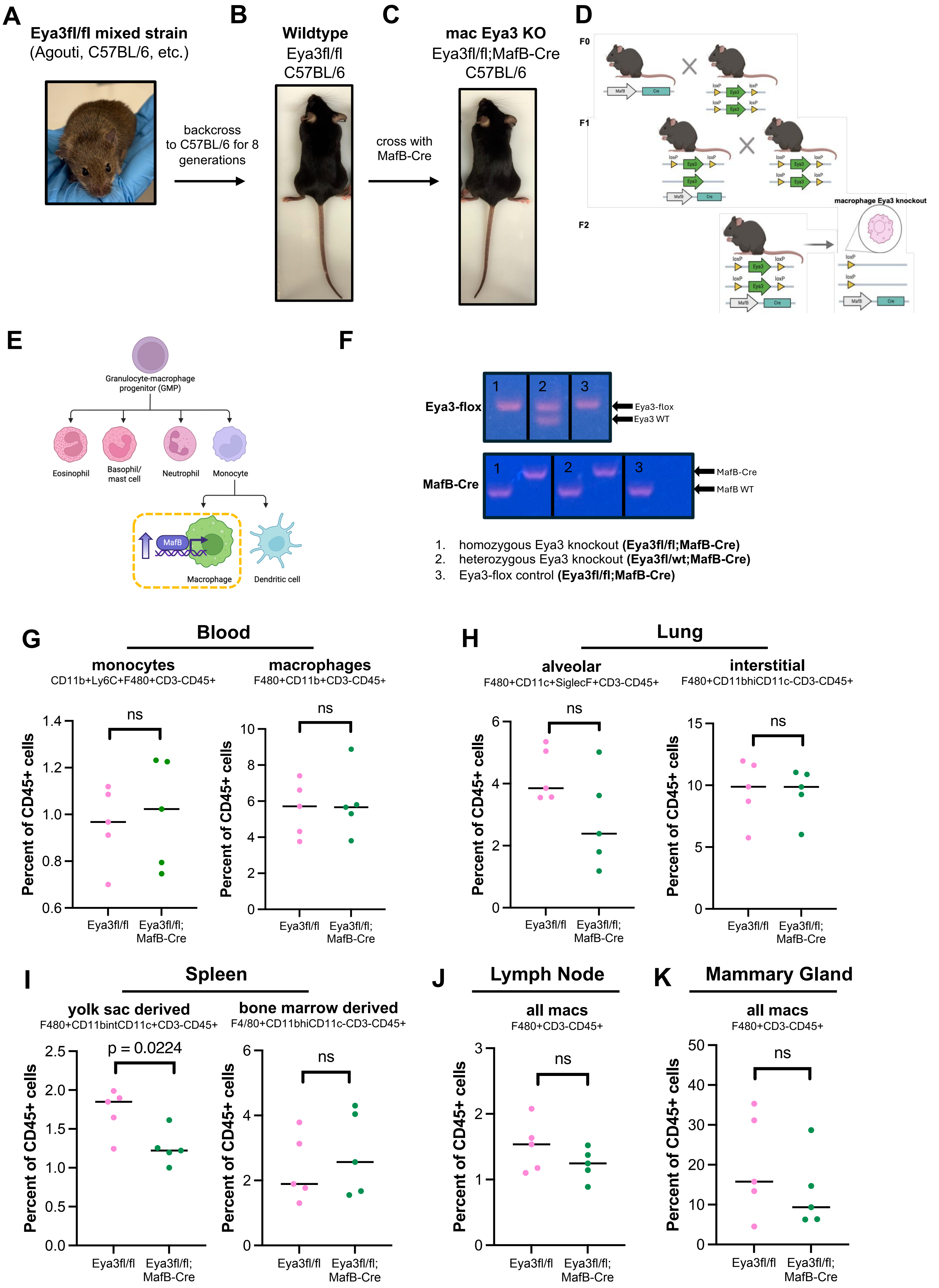

Supplemental Figure 3. *Additional information on the generation and characterization of a C57BL/6 macrophage Eya3 conditional knockout mouse.* A) original Eya3fl/fl mixed strain mouse from R. Hegde lab. B) generation of Eya3fl/fl C57BL/6 mouse through serial backcrossing (8 generations) to C57BL/6 strain C) Eya3fl/fl;MafB-Cre C57BL/6 mouse (macrophage EYA3 knockout). D) complete breeding strategy for homozygous knockout of Eya3 in macrophages with MafB-Cre C57BL/6 and Eya3-flox C57BL/6 mice. E) MafB is upregulated in differentiated macrophages. F) representative PCR products to genotype littermates for Eya3 knockout status. G-K) Flow cytometric characterization of macrophage infiltration of tissues from control (Eya3fl/fl, n = 5) and Eya3 macrophage conditional knockout mice (Eya3fl/fl;MafB-Cre, n = 5). Statistical analysis performed using unpaired t-tests.

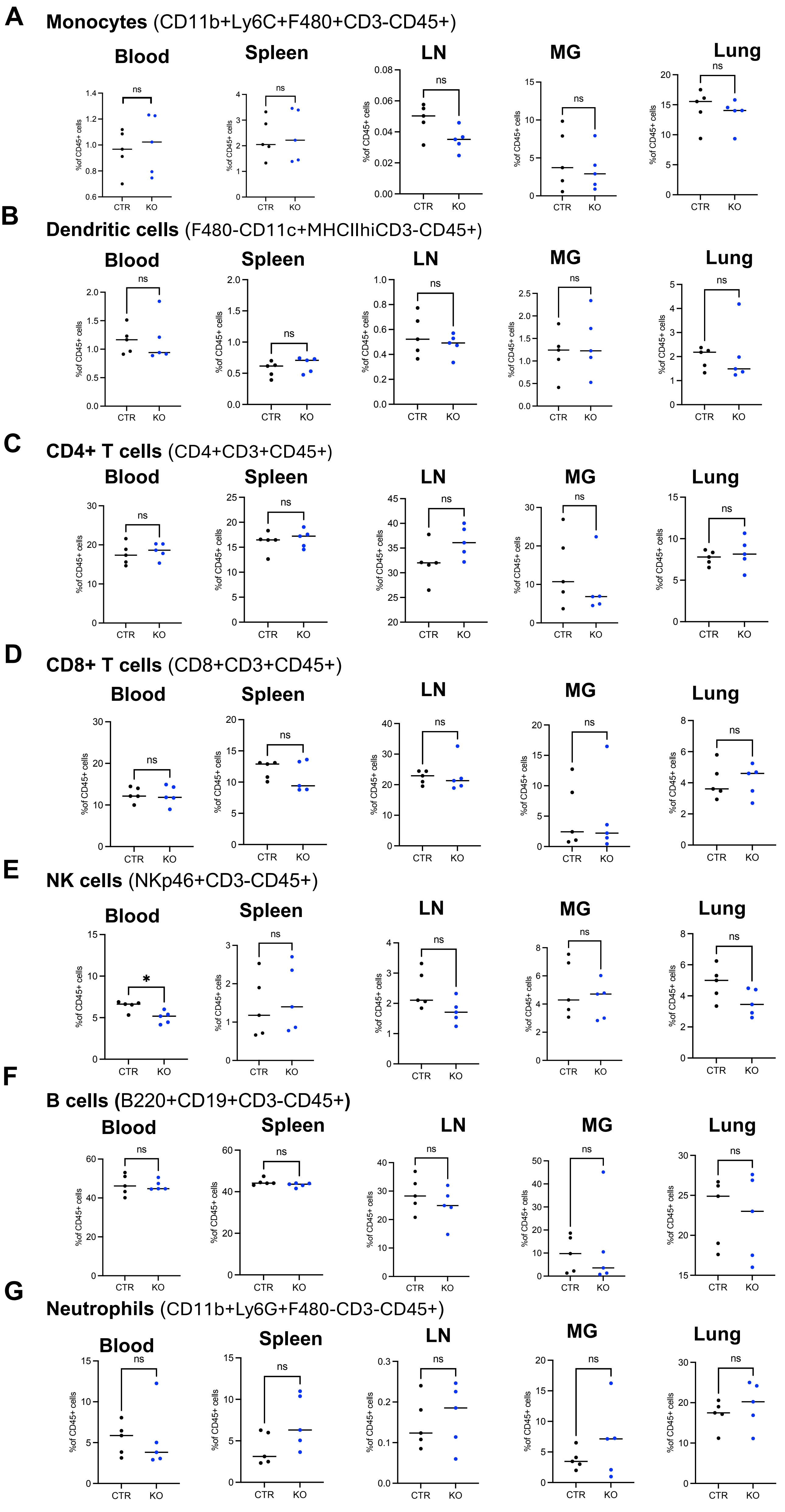

Supplemental Figure 4. ***Quantification of additional immune cell abundance within tissues in Eya3fl/fl (CTR) and Eya3fl/fl;MafB-Cre (KO) mice.*** Flow cytometry analysis to examine: A) monocytes B) Dendritic cells C) CD4+ T cells D) CD8+ T cells E) NK cells F) B cells G) Neutrophils. LN = lymph node, MG = mammary gland from 5 Eya3fl/fl (CTR) and 5 Eya3fl/fl;MafB-Cre (KO) mice. Statistical analysis performed using unpaired t-tests.

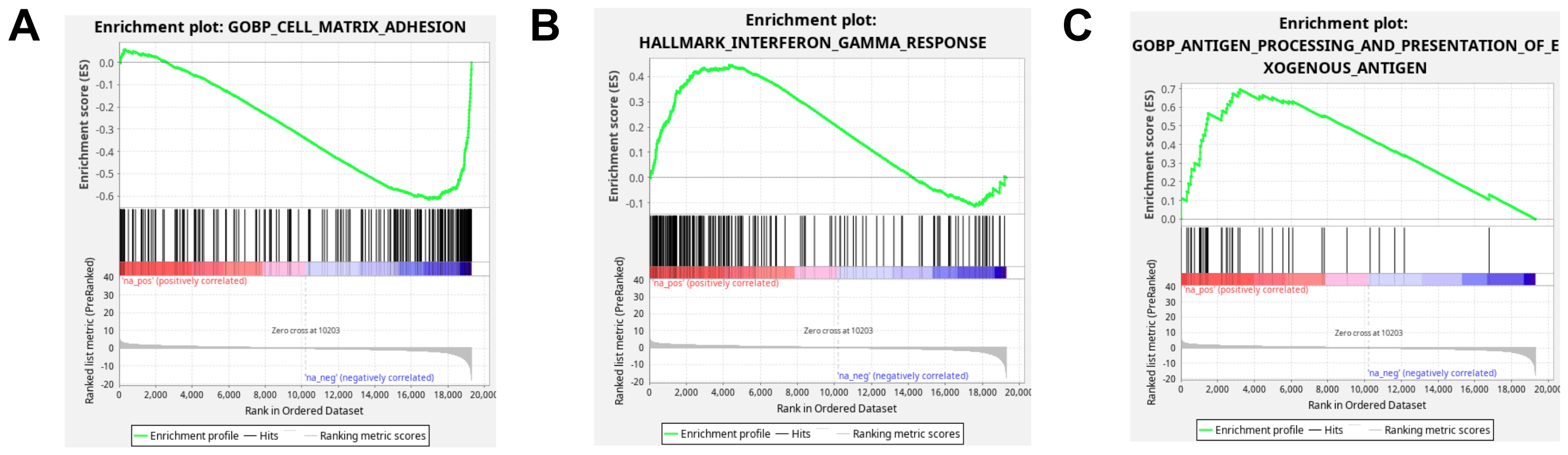

NES = -2.07
FDR q-value = 0.0001

NES = 2.08
FDR q-value = 0.1288

NES = 2.43
FDR q-value = 0.0042

Supplemental Figure 5. ***GSEA plots of pathways relevant to macrophage function from BMDM WT (Eya3fl/fl) and EYA3 KO (Eya3fl/fl;MafB-Cre) bulk RNA-seq.*** GSEA plots comparing BMDM EYA3 KO vs CTR RNA expression: A) Cell Matrix Adhesion B) Interferon Gamma Response E) Interferon Alpha Response C) Antigen Processing and Presentation of Exogenous Antigen

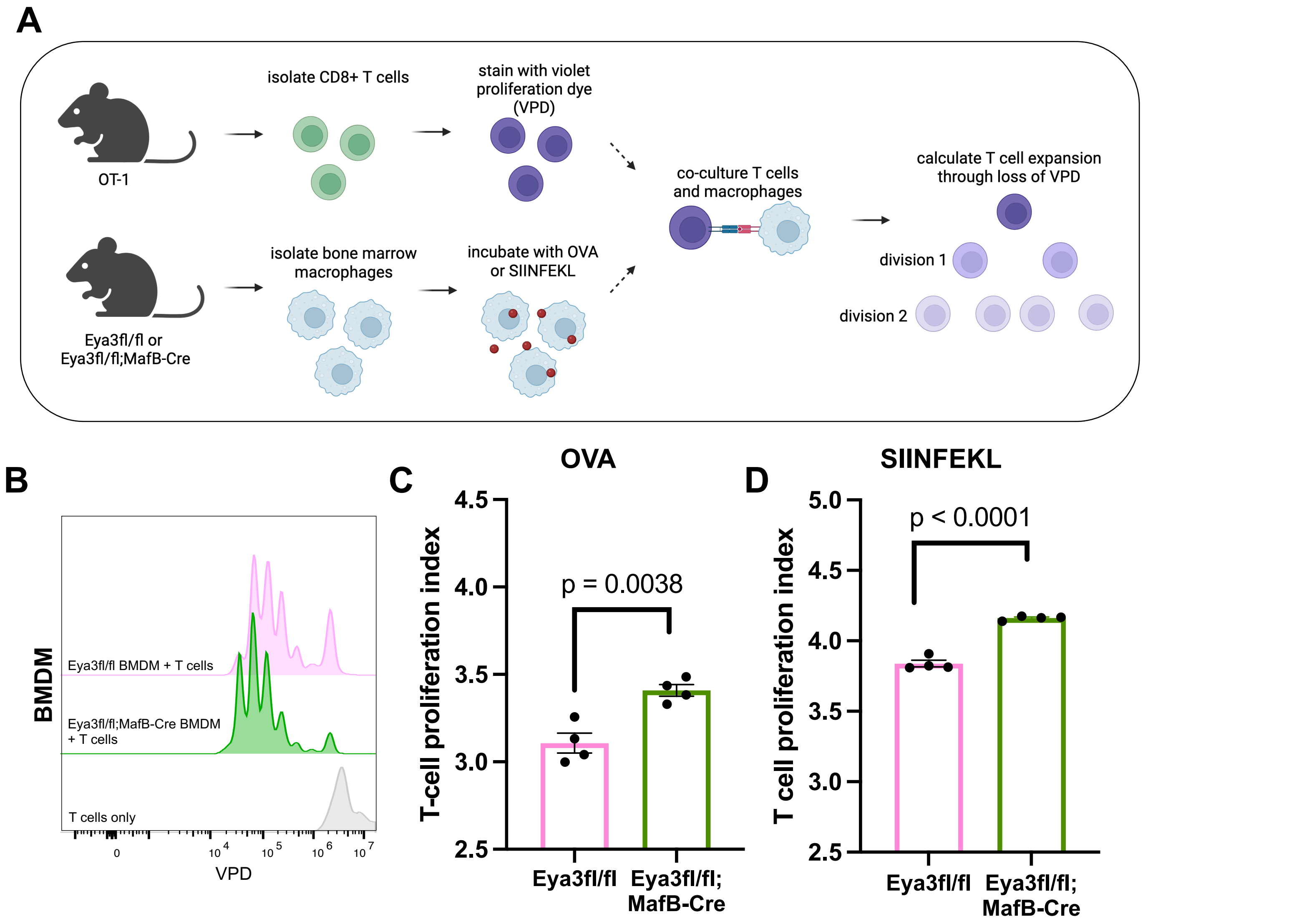

Supplemental Figure 6. *Loss of Eya3 modestly, but significantly, increases antigen presentation in BMDMs ex vivo*. A) Schematic of antigen presentation assay. B) Representative histogram of flow cytometry from co-cultured CD8+CD44+B220- T cells used to evaluate VPD dilution. C) T cell proliferation index when BMDMs are primed with OVA. D) T cell proliferation index (calculated from VPD flow cytometry peaks) when BMDMs are primed with SIINFEKL. Statistical analysis performed using unpaired t-test. Experiments are representative of at least three biological replicates with technical triplicates.

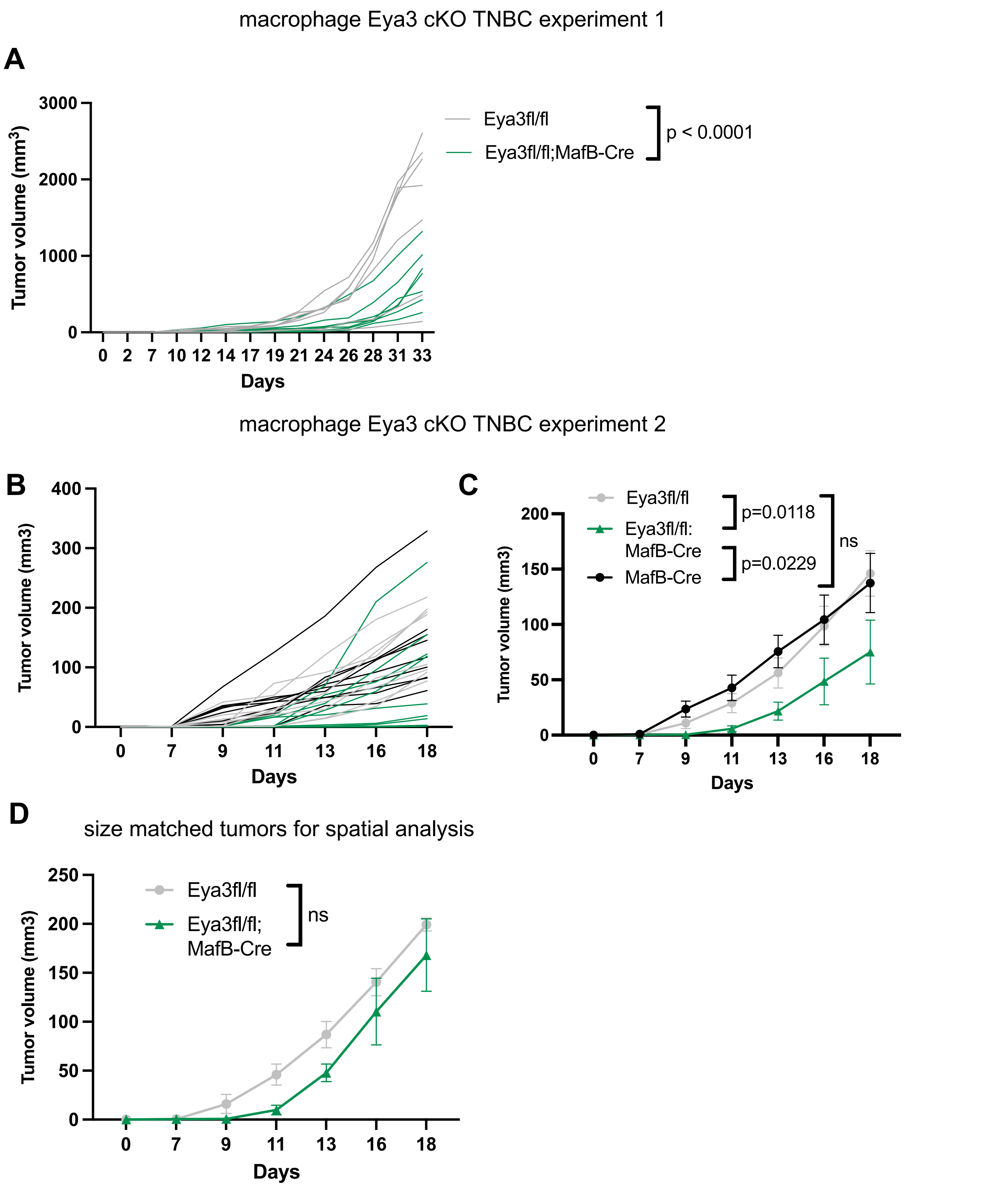

Supplemental Figure 7. ***E0771 tumor growth in macrophage Eya3 conditional knockout mice.*** A) Macrophage Eya3 conditional knockout TNBC experiment 1: tumor growth tracks for individual Eya3fl/fl mice (n = 7) and individual Eya3fl/fl;MafB-Cre mice (n = 7). B-D) Macrophage Eya3 conditional KO TNBC experiment 2: B) tumor growth tracks for individual mice C) Summary data for all 3 groups studied: Eya3fl/fl (Flox CTR, n = 8), MafB-Cre (Cre CTR, n = 9), Eya3fl/fl;MafB-Cre (Eya3 mac KO n = 10). D) Summary tumor growth curves for size matched tumors (n = 4 for each group) analyzed in IHC spatial analysis. Statistical analysis performed using 2-way ANOVA.

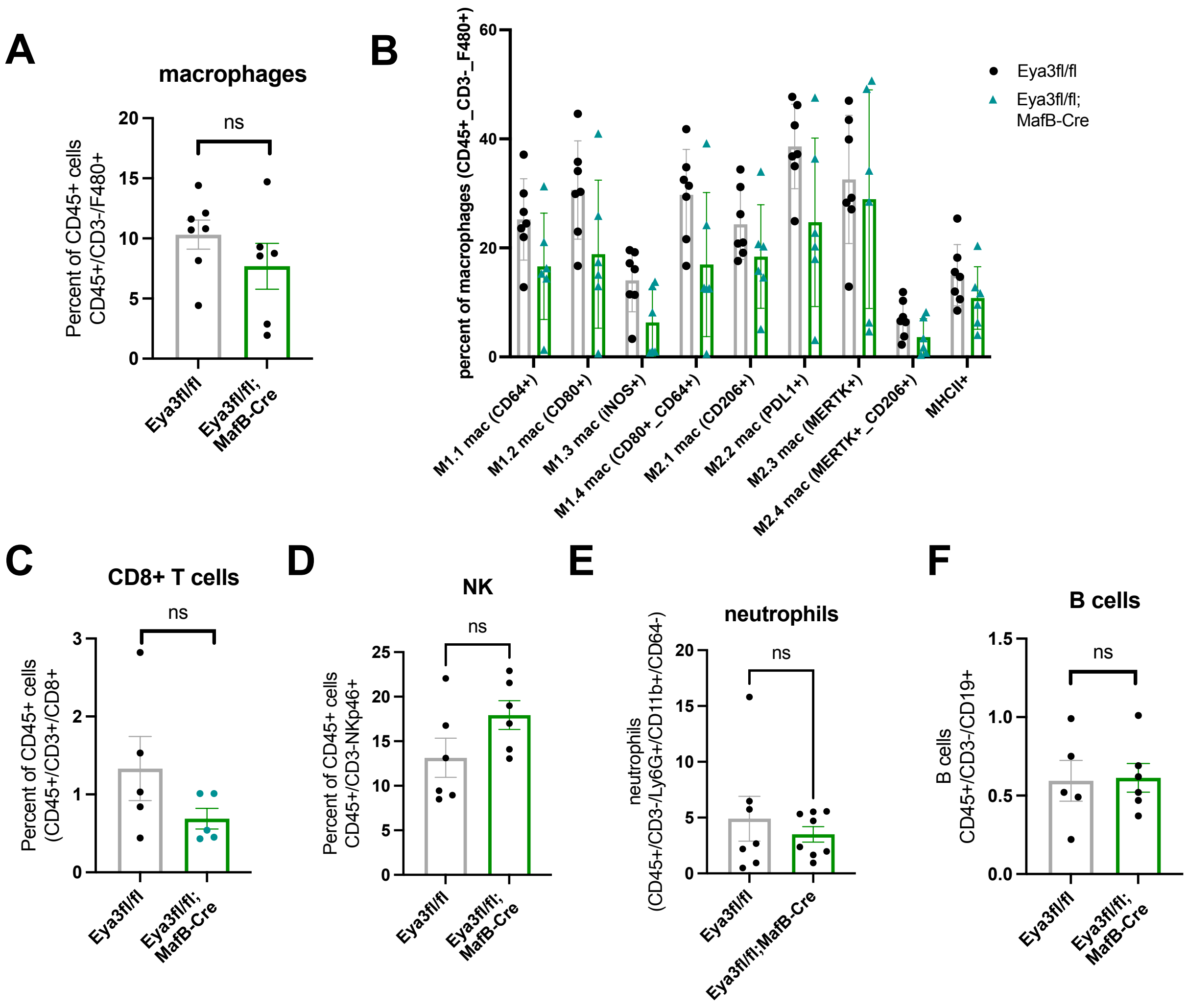

Supplemental Figure 8. ***Flow cytometric analysis of immune infiltration of tumors from Eya3fl/fl or Eya3fl/fl;MafB-Cre mice .*** Analysis performed on tumor experiment #1. A) total macrophages B) Percent of macrophage subtypes C) CD8+ T cells D) NK cells E) neutrophils F) B cells. Statistical analysis performed using unpaired t-tests.

Supplementary Table 1. ***Matrix of IHC markers used to identify different cell populations.*** Matrix used in combination with CELESTA for identification of cell populations within the tumor immune microenvironment.

Subpopulation	Lineage level	DAPI	CD163	CD3	CD80	CD8	EGFR	F480
Mammary fat pad	1_0_1	1	0	0	0	0	NA	0
General Macrophages	1_0_2	1	0	0	NA	0	NA	1
M1 Macrophages	1_0_3	1	0	0	1	0	NA	1
M2 Macrophages	1_0_4	1	1	0	1	0	NA	1
CD3+ T cells	1_0_5	1	0	1	0	0	0	NA
CD8+ T cells	1_0_6	1	0	1	0	1	0	NA
Cancer	1_0_7	1	0	0	NA	0	NA	NA
Hybrid Macrophage	1_0_8	1	NA	0	NA	0	1	1

Supplementary Table 2. ***Expression Thresholds for EYA3 KO CELESTA.*** Matrix of thresholds for analysis of tumors from Eya3fl/fl and Eya3fl/fl;MafB-Cre mice (Fig. 4, Supp. Fig 13).

		Mammary fat pad	General Macrophages	M1 Macrophages	M2 Macrophages	CD3+ T cells	CD8+ T cells	Cancer	Hybrid Macrophage
**Control_3_1**	high_expression_threshold_anchor	0.2	0.6	0.6	0.7	0.6	0.7	0.6	0.6
	high_expression_threshold_index	0.1	0.2	0.2	0.2	0.2	0.2	0.2	0.2
**Control_3_2**	high_expression_threshold_anchor	0.2	0.6	0.6	0.7	0.6	0.7	0.6	0.6
	high_expression_threshold_index	0.1	0.2	0.2	0.2	0.2	0.2	0.2	0.2
**Control_4_1**	high_expression_threshold_anchor	0.2	0.6	0.6	0.7	0.6	0.7	0.6	0.6
	high_expression_threshold_index	0.1	0.2	0.2	0.2	0.2	0.2	0.2	0.2
**Control_4_2**	high_expression_threshold_anchor	0.2	0.6	0.6	0.7	0.6	0.7	0.6	0.6
	high_expression_threshold_index	0.1	0.2	0.2	0.2	0.2	0.2	0.2	0.2
**Control_5_1**	high_expression_threshold_anchor	0.2	0.6	0.6	0.7	0.6	0.7	0.6	0.6
	high_expression_threshold_index	0.1	0.2	0.2	0.2	0.2	0.2	0.2	0.2
**Control_5_2**	high_expression_threshold_anchor	0.2	0.6	0.6	0.7	0.6	0.7	0.6	0.6
	high_expression_threshold_index	0.1	0.2	0.2	0.2	0.2	0.2	0.2	0.2
**Control_6_1**	high_expression_threshold_anchor	0.2	0.6	0.6	0.7	0.6	0.7	0.7	0.6
	high_expression_threshold_index	0.1	0.2	0.2	0.2	0.2	0.2	0.2	0.2
**Control_6_2**	high_expression_threshold_anchor	0.2	0.6	0.6	0.7	0.6	0.7	0.7	0.6
	high_expression_threshold_index	0.1	0.2	0.2	0.2	0.2	0.2	0.2	0.2
**Eya3_cKO_2_1**	high_expression_threshold_anchor	0.2	0.6	0.6	0.6	0.6	0.5	0.6	0.6
	high_expression_threshold_index	0.1	0.2	0.2	0.2	0.2	0.2	0.2	0.2
**Eya3_cKO_2_2**	high_expression_threshold_anchor	0.2	0.6	0.6	0.6	0.6	0.5	0.6	0.6
	high_expression_threshold_index	0.1	0.2	0.2	0.2	0.2	0.2	0.2	0.2
**Eya3_cKO_7_1**	high_expression_threshold_anchor	0.2	0.6	0.6	0.7	0.6	0.5	0.6	0.6
	high_expression_threshold_index	0.1	0.2	0.2	0.2	0.2	0.2	0.2	0.2
**Eya3_cKO_7_2**	high_expression_threshold_anchor	0.2	0.6	0.6	0.6	0.6	0.5	0.6	0.6
	high_expression_threshold_index	0.1	0.2	0.2	0.2	0.2	0.2	0.2	0.2
**Eya3_cKO_8_1**	high_expression_threshold_anchor	0.2	0.6	0.6	0.6	0.6	0.5	0.7	0.6
	high_expression_threshold_index	0.1	0.2	0.2	0.2	0.2	0.2	0.3	0.2
**Eya3_cKO_8_2**	high_expression_threshold_anchor	0.2	0.6	0.6	0.6	0.6	0.5	0.7	0.6
	high_expression_threshold_index	0.1	0.2	0.2	0.2	0.2	0.2	0.3	0.2
**Eya3_cKO_9_1**	high_expression_threshold_anchor	0.2	0.6	0.6	0.6	0.5	0.5	0.6	0.6
	high_expression_threshold_index	0.1	0.2	0.2	0.2	0.2	0.2	0.2	0.2
**Eya3_cKO_9_2**	high_expression_threshold_anchor	0.2	0.6	0.6	0.7	0.6	0.5	0.6	0.6
	high_expression_threshold_index	0.1	0.2	0.2	0.2	0.2	0.2	0.2	0.2

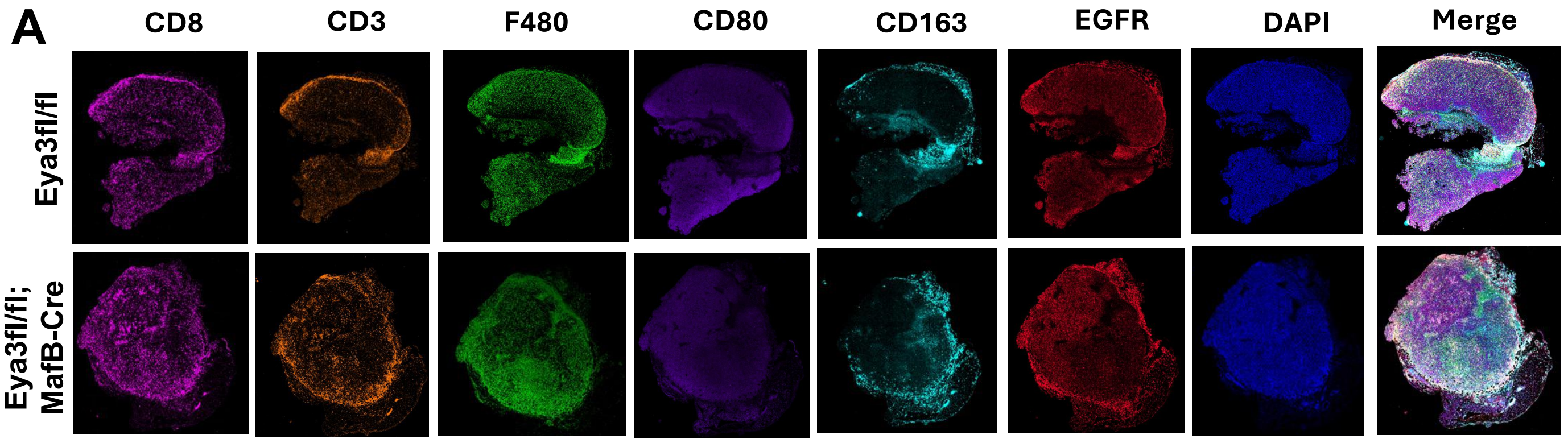

B

	CD163	CD80	EGFR	F480
Gen Mac	0	175	10-20	200-300
M1	0	200-400	10-50	175-250
M2	75-100	150-200	10-50	150-250
Cancer/Mac?	25-50	175	75-100	150-200

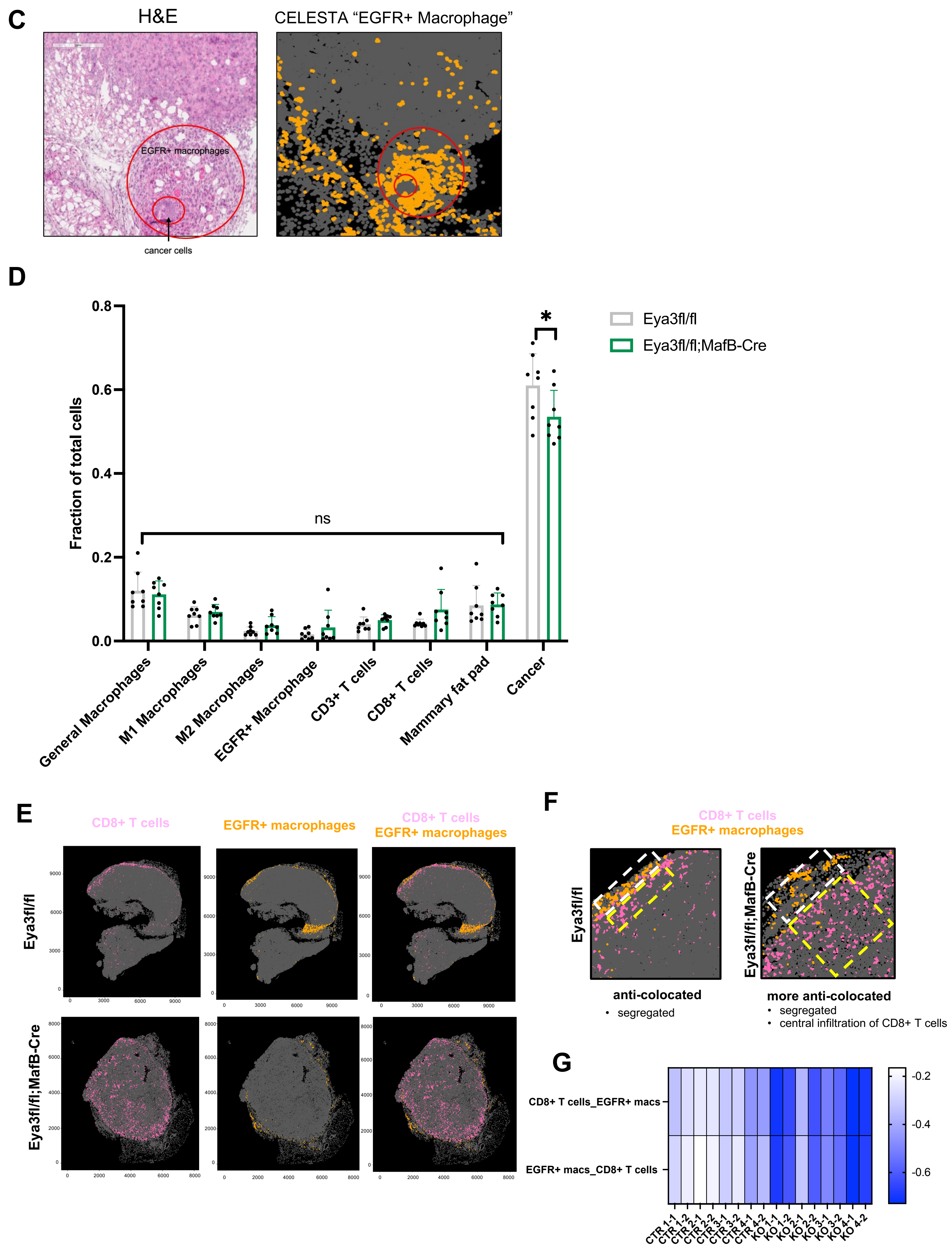

Supplemental Figure 9. ***Supplementary material for spatial analysis of tumors from Eya3fl/fl and Eya3fl/fl;MafB-Cre mice.*** A) Fluorescent IHC images of all markers used to identify cell populations through CELESTA B) Expression ranges of macrophage markers; unexpected macrophage population identified that expresses tumor marker "EGFR". C) H&E staining demonstrating that the EGFR+ population is a macrophage subpopulation. Population was termed "EGFR+ macrophage ". D) Cell populations represented as fraction of total cells. Statistical analysis performed using unpaired t-tests. E) CELESTA plots of CD8+ T cells and EGFR+ macrophages. F) Zoomed in representation of increased anti-colocation of CD8+ T cells and EGFR+ macrophages in tumors. G) Normalized CLQs between CD8+ T cells and EGFR+ macrophages.

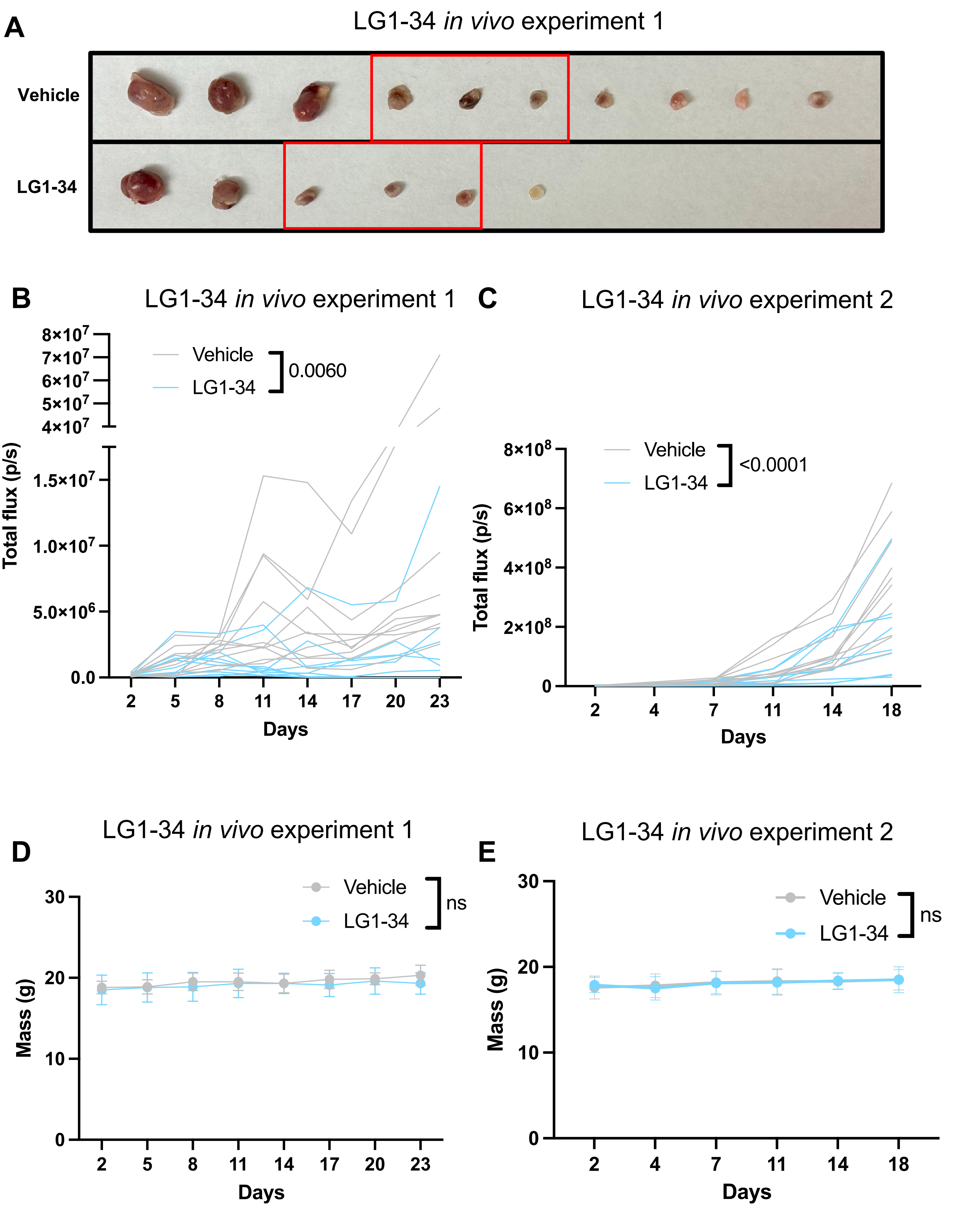

Supplemental Figure 10. ***Additional plots for vehicle vs LG1-34 TNBC tumor experiments.*** A) LG1-34 TNBC exp 1: Tumors resected on day 23; complete response was observed in 4/10 tumors in LG1-34 treated group, thus only the 6 left-most tumors per group were analyzed via IHC-based spatial analysis. Red boxes surround "size-matched small" tumors analyzed in Fig. 6F, I. B) tumor growth tracks for individual mice (vehicle n = 10, LG1-34 n = 10) from LG1-34 experiment 1. C) tumor growth tracks for individual mice (vehicle n = 10, LG1-34 n = 10) from LG1-34 experiment 2. Mouse weights over time for LG1-34 experiment 1 (D) and 2 (E). Statistical analysis was performed using 2way-ANOVA.

Supplementary Table 3. ***Expression Thresholds for LG1-34 CELESTA.*** Matrix of thresholds for analysis of tumors from C57BL/6 mice treated with vehicle or LG1-34 (Fig. 6, Supp. Fig 16).

		Mammary fat pad	General Macrophages	M1 Macrophages	M2 Macrophages	CD3+ T cells	CD8+ T cells	Cancer	Hybrid Macrophage
**Vehicle_1**	high_expression_threshold_anchor	0.7	0.8	0.4	0.4	0.5	0.4	0.3	0.6
	high_expression_threshold_index	0.7	0.3	0.2	0.2	0.2	0.2	0	0.2
**Vehicle_2**	high_expression_threshold_anchor	0.7	0.8	0.4	0.4	0.5	0.4	0.3	0.6
	high_expression_threshold_index	0.7	0.3	0.2	0.2	0.2	0.2	0	0.2
**Vehicle_3**	high_expression_threshold_anchor	0.7	0.8	0.4	0.4	0.4	0.7	0.3	0.6
	high_expression_threshold_index	0.7	0.3	0.2	0.2	0.2	0.2	0	0.2
**Vehicle_4**	high_expression_threshold_anchor	0.7	0.8	0.4	0.4	0.5	0.4	0.3	0.6
	high_expression_threshold_index	0.7	0.3	0.2	0.2	0.2	0.2	0	0.2
**Vehicle_5**	high_expression_threshold_anchor	0.7	0.8	0.4	0.4	0.4	0.7	0.3	0.6
	high_expression_threshold_index	0.7	0.3	0.2	0.2	0.2	0.2	0	0.2
**Vehicle_6**	high_expression_threshold_anchor	0.7	0.8	0.4	0.4	0.5	0.4	0.3	0.6
	high_expression_threshold_index	0.7	0.3	0.2	0.2	0.2	0.2	0	0.2
**LG1-34_1**	high_expression_threshold_anchor	0.7	0.8	0.4	0.4	0.5	0.4	0.3	0.6
	high_expression_threshold_index	0.7	0.3	0.2	0.2	0.2	0.2	0	0.2
**LG1-34_2**	high_expression_threshold_anchor	0.7	0.8	0.4	0.4	0.4	0.7	0.3	0.6
	high_expression_threshold_index	0.7	0.3	0.2	0.2	0.2	0.2	0	0.2
**LG1-34_3**	high_expression_threshold_anchor	0.7	0.8	0.4	0.4	0.4	0.7	0.3	0.6
	high_expression_threshold_index	0.7	0.3	0.2	0.2	0.2	0.2	0	0.2
**LG1-34_4**	high_expression_threshold_anchor	0.7	0.8	0.4	0.4	0.5	0.4	0.3	0.6
	high_expression_threshold_index	0.7	0.3	0.2	0.2	0.2	0.2	0	0.2
**LG1-34_5**	high_expression_threshold_anchor	0.7	0.8	0.4	0.4	0.5	0.4	0.3	0.6
	high_expression_threshold_index	0.7	0.3	0.2	0.2	0.2	0.2	0	0.2
**LG1-34_6**	high_expression_threshold_anchor	0.7	0.8	0.4	0.4	0.4	0.7	0.3	0.6
	high_expression_threshold_index	0.7	0.3	0.2	0.2	0.2	0.2	0	0.2

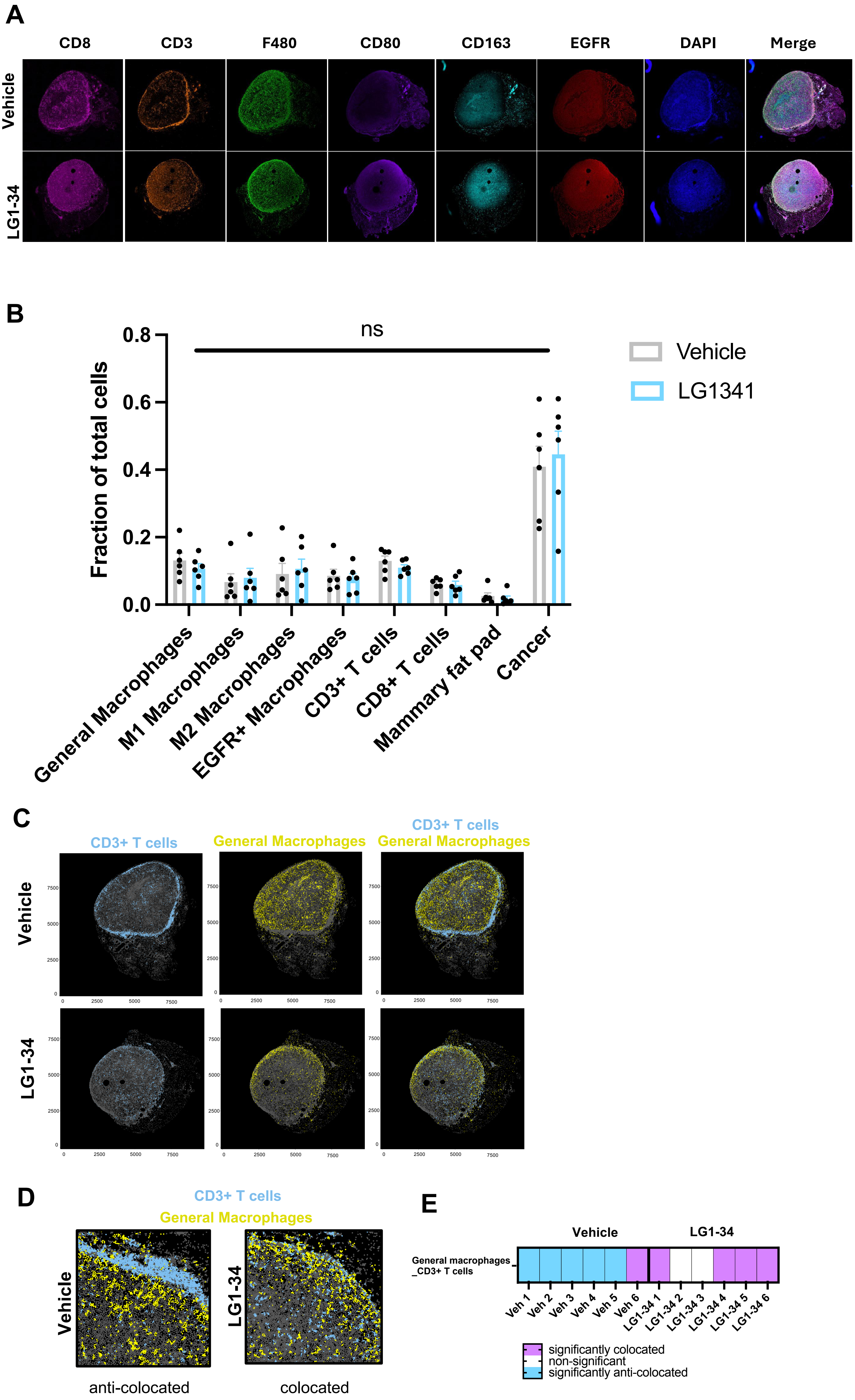

Supplemental Figure 11. ***Supplementary material for spatial analysis of LG1-34 treated tumors.*** A) Fluorescent IHC images of all markers used to identify cell populations through CELESTA B) Cell populations represented as fraction of total cells. Statistical analysis performed using t-tests. C) CELESTA plots of CD3+ T cells and general macrophages. D) Zoomed in representation of colocation between CD3+ T cells (CD3+/CD8-) and general macrophages (F480+ only) in tumors from vehicle and LG1-34 treated mice. E) Colocation analysis of general macrophages and CD3+ T cells in tumors from vehicle and LG1-34 treated mice. Purple = significant colocation within a sample, white = non-significant, blue = significant anti-colocation within a sample. Significance assessed through spatial permutation analysis.

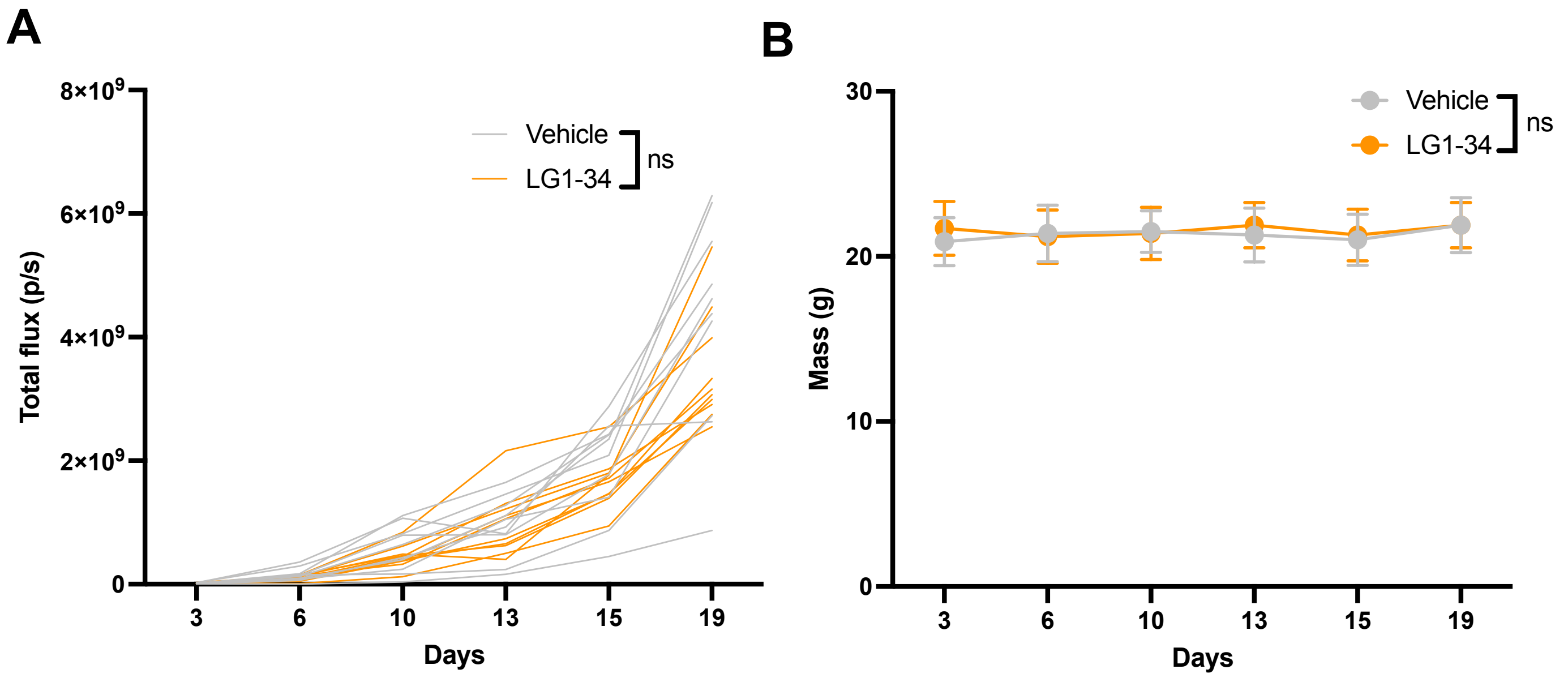

Supplemental Figure 12. ***Supplementary plots for Vehicle vs LG1-34 TNBC experiment in immune compromised animals.*** *A*) tumor growth tracks for individual mice from Fig. 6K (vehicle n = 10, LG1-34 n = 10). E) Mouse weights over time. Statistical analysis was performed using 2way-ANOVA.

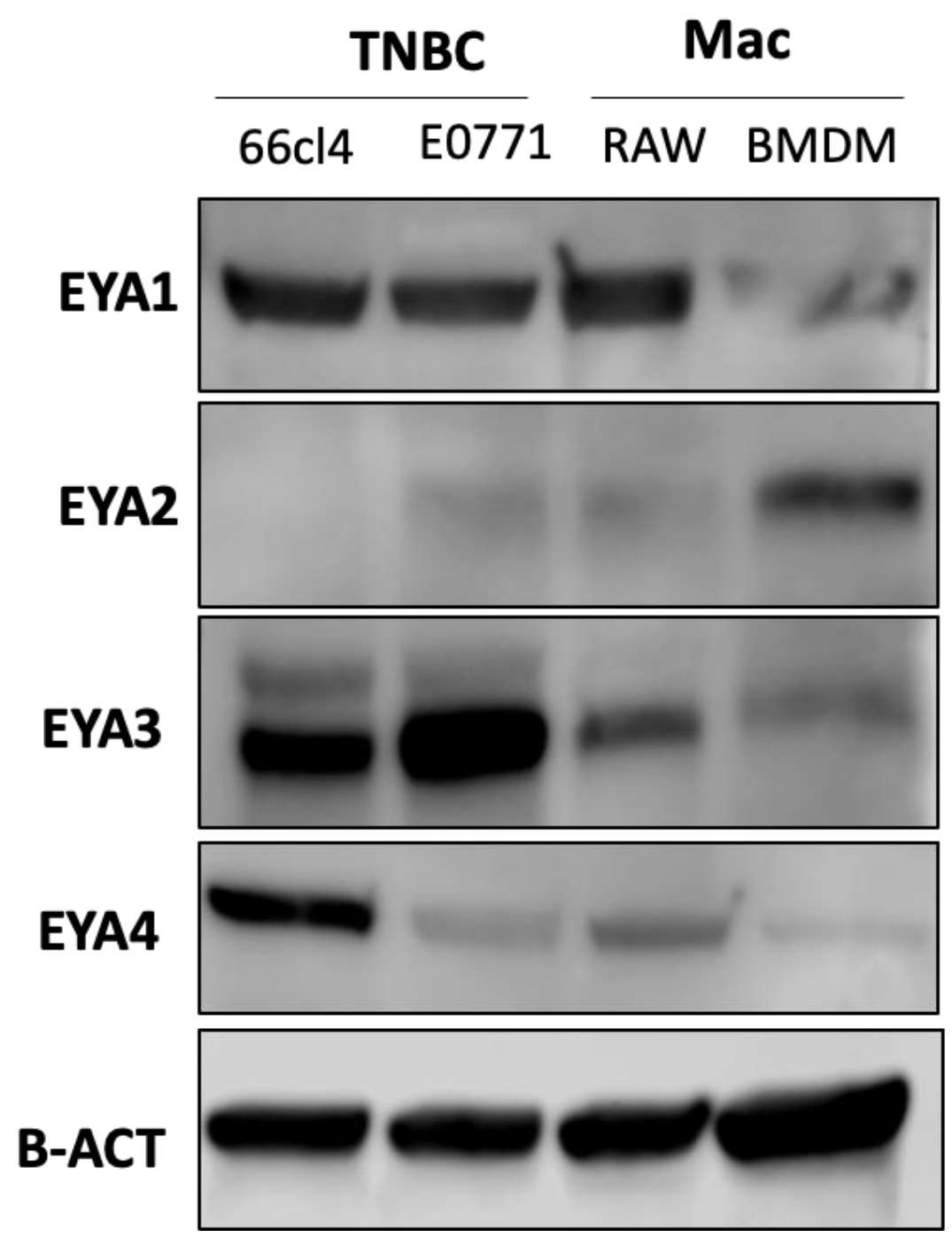

Supplemental Figure 13. ***Eyas1-4 in TNBC and macrophages.*** Western blot analysis of Eya1-4 protein expression in TNBC cell lines 66cl4 and E0771, and macrophage cell line RAW264.7, as well as bone marrow derived macrophages (BMDM) from whole cell lysate.

Supplementary Table 4. ***Primary and secondary antibody reagent information.*** All primary and secondary antibodies and pertinent information used in Western blot assays**.**

Primary antibody	Source	Identifier	Clone Number (if Applicable)
Rabbit polyclonal anti-EYA1	Proteintech	22658-1-AP	N/A
Rabbit monoclonal anti-EYA2	Invitrogen	MA5-52842	23GB5350
Rabbit polyclonal anti-EYA3	Bethyl	A302-689A	N/A
Mouse monoclonal anti-EYA4	Santa Cruz Biotech	sc-393111	E-11
Rabbit monoclonal anti-GAPDH	Cell Signaling Technology	2118S	14C10
Mouse monoclonal anti-β-Actin	Sigma-Aldrich	A5316	AC-74
Goat anti-mouse HRP Secondary antibody	LICORbio	926-80010	N/A
Goat anti-rabbit HRP secondary antibody	LICORbio	92680011	N/A

Supplementary Table 5. ***Flow cytometry antibodies.*** Antibody panels used for flow cytometry analysis of tissues or cells

T and B cell panel

	Marker	Color	Catalog no.	Clone
1	Zombie Live Dead	Aqua	423101	
2	CD45	BV421	103134	30-F11
3	CD3	BV605	100237	17A2
4	CD8a	BV650	100742	53-6.7
5	CD4	PE	ebio	
6	CD19	APC-Cy7	115529	6D5
7	B220	PerCPCy5.5	biol	RA3-6B2

NK cell panel

	Marker	Color	Catalog no.	Clone
1	Zombie Live Dead	Aqua	423101	
2	CD45	BV421	103134	30-F11
3	CD3	BV605	100237	17A2
4	NKp46	PE	137604	29A1.4
5	CD49b	PE-Cy7	108905	DX5
6	CD11b	PerCP Cy5.5	#45-0112-82	M1/70
7	CD27	APC-Cy7	124225	LG.3A10

Innate Immune Panel (some markers switched in and out to maintain panel compatibility)

	Marker	Color	Catalog no.	Clone
1	Zombie Live Dead	Aqua (BV510)	423101	
2	CD45	BV421	103134	30-F11
3	CD3	BV605	100237	17A2
4	F4/80	FITC	123108	BM8
5	Ly6C	BV711	128037	HK1.4
6	Ly6G	AF700	127621	1A8
7	CD11b	PerCP Cy5.5	ebio	M1/70
8	CD11c	BV785	117335	N418
9	MHC-II (I-A/I-E)	APC/ Cy7	107628	M5/114.15.2
10	CD206	PE	141705	C068C2
11	CD80	APC	#17-0801-82	16-10A1
12	PD-L1	PE-Cy7	biol	10F.9G2
13	MERTK	APC	Biol	
14	iNOS	BUV737	367-5920-80	CXNFT
15	CD64	BV605	139323	X54-5/7.1
16	CD80 alt.	BV650	100742	53-6.7
